# Comparison of atherosclerotic cardiovascular disease (ASCVD) and Framingham risk scores (FRS) in an Iranian population

**DOI:** 10.1016/j.ijcrp.2024.200287

**Published:** 2024-05-24

**Authors:** Matin Sepehrinia, Hossein Pourmontaseri, Mehrab Sayadi, Mohammad Mehdi Naghizadeh, Reza Homayounfar, Mojtaba Farjam, Azizallah Dehghan, Abdulhakim Alkamel

**Affiliations:** a Student Research Committee, Fasa University of Medical Sciences, Fasa, Iran; b Noncommunicable Diseases Research Center, Fasa University of Medical Sciences, Fasa, Iran; c Cardiovascular Research Center, Shiraz University of Medical Sciences, Shiraz, Iran; d National Nutrition and Food Technology Research Institute (WHO Collaborating Center), Faculty of Nutrition Sciences and Food Technology, Shahid Beheshti University of Medical Sciences, Tehran, Iran; e Department of Cardiovascular Disease, Faculty of Medicine, Fasa University of Medical Sciences, Fasa, Iran

**Keywords:** Risk factor, Prevention, Risk prediction, Risk assessment, Pooled cohort equation, Iran

## Abstract

**Background:**

Framingham risk score (FRS) and Atherosclerotic Cardiovascular Disease risk score (ASCVDrs) are widely used tools developed based on the American population. This study aimed to compare the ASCVDrs and FRS in an Iranian population.

**Method:**

The participants of the Fasa Adult Cohort Study and the patients of the cardiovascular database of Vali-Asr Hospital of Fasa, aged 40–80 years, were involved in the present cross-sectional study. After excluding non-eligible participants, the individuals with a history of myocardial infarction or admission to the cardiology ward due to heart failure were considered high-risk, and the others were considered low-risk. The discriminative ability of FRS and ASCVDrs was evaluated and compared using receiver operating characteristic curve analysis. The correlation and agreement of ASCVDrs and FRS were tested using Cohen Kappa and Spearman.

**Results:**

Finally, 8983 individuals (mean age:53.9 ± 9.5 y, 49.2 % male), including 1827 high-risk participants, entered the study. ASCVDrs detected a greater portion of participants as high-risk in comparison with FRS (28.7 % vs. 15.7 %). ASVD (AUC:0.794) had a higher discriminative ability than FRS (AUC:0.746), and both showed better discrimination in women. Optimal cut-off points for both ASCVDrs (4.36 %) and FRS (9.05 %) were lower than the original ones and in men. Compared to FRS, ASCVDrs had a higher sensitivity (79.3 % vs. 71.6 %) and lower specificity (64.5 % vs. 65.1 %). FRS and ASCVDrs had a moderate agreement (kappa:0.593,*p*-value<0.001) and were significantly correlated (Spearman:0.772,*p*-value<0.001).

**Conclusions:**

ASCVDrs had a more accurate prediction of cardiovascular events and identified a larger number of people as high-risk in the Iranian population.

## Highlights

• ASCVD risk score had a higher discriminative ability and identified more people as high-risk than FRS among Iranians.
• Both risk scores had higher discrimination ability in women.
• Both ASCVD and FRS had an acceptable performance and moderate agreement among Iranians.
• Risk prediction models have different performances across populations because of the differences in lifestyle and genetics.

## 1 Introduction

Despite magnificent progress in prevention and treatment, the prevalence of cardiovascular disease (CVD) has doubled during the past three decades [1]. In 2022, CVD caused approximately 19.8 million deaths worldwide, accounting for 34.9 % of global all-cause mortality. Also, CVD had the highest age-standardized disability-adjusted life years (DALYs) among all diseases with nearly 5078.4 per 100,000 individuals globally [2]. CVD imposes a significant health burden in Iran as well. In 2015, more than 9000 people in every 100,000 Iranians were suffering from CVD, which led to 46 % of all-cause mortality [3]. A recent study revealed that more than 50 % 0f 10-year CVD incidence and nearly 20 % of CVD mortality were attributed to the five modifiable CVD risk factors including smoking, obesity, dyslipidemia, hypertension, and diabetes [4]. Insufficient CVD risk control is a major problem in public health, and prevention strategies should be undertaken seriously [5].

Risk assessment is the first step for primary prevention that helps physicians to determine the individuals who benefit the most from preventive interventions, like prescribing lipid-lowering drugs, in a personalized manner [6]. Many risk scores, such as Framingham Risk Score (FRS) [7], Atherosclerotic Cardiovascular Disease risk score (ASCVDrs) [8], Systemic Coronary Risk Evaluation (SCORE) [9], and World Health Organization/International Society of Hypertension CVD risk prediction charts (WHO) [10] have been developed to predict the CVD probability and identify the high-risk individuals. FRS and ASCVDrs are the most well-known risk scores worldwide and have been validated externally in many populations and ethnic groups [[11], [12], [13]].

Framingham Risk Score (FRS) was the first risk assessment tool for CVD prevention, designed based on the Framingham study. Very soon, FRS grabbed so much attention and found its place in the primary prevention guidelines, such as Adult Treatment Panel (ATP) III. Over the years, FRS has been developed several times. In 2008, the Framingham Risk Score was developed to predict global CVD (including coronary heart diseases (CHD), cerebrovascular disease, intermittent claudication, and congestive heart failure) [7,13].

In 2013, the American College of Cardiology and the American Heart Association (ACC/AHA) introduced ASCVDrs as the first sex- and race-specific risk score. The ASCVDrs was developed based on the data from participants with at least 12 years of follow-up in several cohorts in the United States. ASCVDrs was designed to predict the 10-year risk for developing the first atherosclerotic cardiovascular event in African-American and white individuals aged between 40 and 79 [8]. Risk assessment using the ASCVDrs is currently the cornerstone of the ACC/AHA guideline for primary prevention [14].

Both ASCVDrs and FRS were developed based on the data from the cohorts conducted in the United States. Since clinical decision-making for preventive intervention depends on the risk assessment models, it is important to choose the one with the most accurate prediction ability for the target population [15]. Several studies have compared these two risk scores in different countries, such as India [16] and Australia [17]. However, the comparison of ASCVDrs and FRS in the Iranian population is scarce [18]. The present study aimed to compare the discrimination ability of ASCVDrs and FRS and find the appropriate cut-off for each one in the south of Iran.

## 2 Methods

### 2.1 Study design

This cross-sectional study was performed on a population combined from the Adult Cohort Study (FACS) [19] and the Fasa Registry on Systolic Heart Failure (FaRSH) [20]. FACS included 10139 individuals invited from Sheshdeh (a village of Fasa, Iran) to fill out the Questionnaires asking for detailed information about baseline features, medical history, and anthropometric assessments. The first phase of this study was completed in 2016, and follow-up of included participants has been continued till the conduction of the present study [19]. FaRSH is another database that was collected in the Cardiology Ward of Vali-Asr Hospital, Fasa, which was performed under the observation of the Fasa University of Medical Science. This study included the participants who were admitted with Heart Failure Events and followed them for one-, six- and twelve-month periods after admission. Till now, more than 2500 patients have been included in this study, and a detailed Web-based Questionnaire, including baseline features, clinical condition (especially details about cardiological information), and paraclinical data, were collected. The participants aged 40–80 years were included. Then, the participants with outlier and missing data, as well as those who were not sure about their medical history, were excluded from the study [20].

### 2.2 Measurements

Age (year), gender (men, women), smoking (yes/no), cholesterol (mg/dL), high-density lipoprotein (HDL, mg/dL), and systolic blood pressure (SBP, mmHg) were collected based on recorded databases. In the case of medical history, including diabetes, hypertension, and myocardial infarction, the recorded database was matched with electronic documents, medications, and the self-report of participants to achieve the most accurate data collection.

In this study, the cardiac event was defined as myocardial infarction and heart failure. Participants with a history of cardiac events were allocated to the high-risk group and the remaining participants were allocated to Low-risk groups. The participants who were admitted to the cardiology ward in FaRSH because of acute heart failure, alongside the participants with a history of myocardial infarction, were included in the high-risk group. The risk score with a higher ability to discriminate between high and low-risk individuals has a better performance.

The Framingham Risk Score (FRS) is a scale to assess the risk of 10-year Cardiac events. In this scale, cardiometabolic risk factors, including age, gender, SBP, HDL, cholesterol, and smoking, were assessed to categorize and score each one. Then, the scores were summed, and the total FRSs of evaluated individuals were obtained. Eventually, these scores were converted to matched percentages, which show the risk of cardiac events in the next 10 years [7].

The ASCVDrs is the other cardiac event risk score that was applied in the present study. ASCVDrs is a 10-year risk score capable of assessing risk in persons aged 40–79 years, specified for sex and race. Different cardiometabolic risk factors, including age, gender, race, HDL, total cholesterol, diabetes, hypertension, and lowering blood pressure medications, were combined to assess ASCVDrs in the version released in 2013 by AHA/ACC. The score of each parameter was computed by a race-sex-specified formula and then summed to achieve a total score for each one. Finally, the survival rate of each sex-race group was powered by the interval of the individual score and the mean score of the population to obtain the chance of a Cardiac event in the next 10 years for each individual [8].

### 2.3 Statistical analysis

The quantitative and qualitative variables were reported as mean (standard deviation) and frequency (percent). The cardiometabolic risk factors were compared among high- and low-risk groups using chi-square and independent T-test. The Receiver Operating Characteristic (ROC) curve analysis was applied to obtain optimal cut-off points for ASCVDrs and FRS in the detection of high-risk groups. Also, the discrimination ability of FRS and ASCVDrs was compared using the area under the curve (AUC) of the ROC curve. Additionally, sensitivity, specificity, negative predictive value (NPV), and positive predictive value (PPV) of the risk score were calculated to compare their performance. All statistical analysis process was performed in SPSS v.16 (IBM Inc., Chicago, Ill). The agreement and correlation of ASCVDrs and FRS were tested using the Cohens Kappa and Spearman test. The significance level was considered as a P value < 0.05.

### 2.4 Ethics approval and consent to participate

The present study was confirmed by the Ethics Committee of Fasa University of Medical Sciences (Approval Code: IR.FUMS.REC.1401.110) and following the Helsinki Declaration. All participants were alerted about the aim of the research and fulfilled the written informed consent.

## 3 Results

After exclusion, 8983 individuals including 1827 (20.3 %) high-risk participants (admitted into the cardiology ward due to heart failure or having a history of myocardial infarction), were studied. The mean age of the studied population was 53.9 ± 9.5 years, containing 4419 (49.2 %) men. Table 1 compares the risk factors of cardiac events among low- and high-risk groups. The frequency and amount of all risk factors were significantly higher in the high-risk group, except for hypertension, which was significantly higher in the low-risk group. The high-risk group had a significantly greater risk of cardiac events based on both ASCVDrs and FRS.

**Table 1:** Comparison of characteristics of studied population among low- and high-risk individuals for cardiac events, means ± SD or N (%).

Variable	Total (N = 8983)	Low-risk N = 7156 (79.7 %)	High-risk N = 1827 (20.3 %)	P value^a^
Gender (male)	4419 (49.2 %)	3291 (46.0 %)	1128 (61.7 %)	<0.001
Age (y)	53.9 ± 9.5	51.7 ± 8.0	62.8 ± 9.8	<0.001
Hypertension	1849 (20.6 %)	1687 (23.6 %)	162 (8.9 %)	<0.001
SBP (mmHg)	115 ± 20	113 ± 19	123 ± 22	<0.001
MI	1342 (14.9 %)	0 (0 %)	1342 (73.5 %)	<0.001
DM	1607 (17.9 %)	1036 (14.5 %)	571 (31.3 %)	<0.001
Smoking	2499 (27.8 %)	2030 (28.4 %)	469 (25.7 %)	0.022
Cholesterol (mg/dl)	178 ± 40	184 ± 36	151 ± 41	<0.001
HDL (mg/dl)	46.8 ± 11.6	48.5 ± 11.1	40.0 ± 10.9	<0.001
ASCVDrs	6.62 ± 8.2	4.8 ± 5.7	13.8 ± 11.8	<0.001
FRS	10.8 ± 9.84	9.1 ± 8.2	17.5 ± 12.5	<0.001

High-risk, including persons with a history of myocardial infarction OR admitted to the cardiology ward of Vali-Asr hospital due to heart failure; SBP, systolic blood pressure; MI, Myocardial Infarction; DM, Diabetes Mellitus; HDL, High-density lipoprotein; ASCVDrs, Atherosclerotic Cardiovascular Disease Risk Score; FRS, Framingham Risk Score.

^a^The qualitative (frequency and percent) variables were compared using chi-square. The Quantitative (mean and Standard Deviation) variables were compared using Independent T-test except for ASCVDrs and FRS (their distribution was not normal and were compared using Mann-Whitney).

^b^The quantitative variables were reported as Mean ± Standard Deviation. The qualitative variables were reported as frequency (percent).

Table 2 shows the risk for 10-year cardiac events assessed by FRS and ASCVDrs. Among the studied population, ASCVDrs considered more individuals as high risk than FRS significantly, 2576 (28.7 %) vs. 1407 (15.7 %), *p*-value <0.001. Additionally, our findings showed that FRS and ASCVDrs had a moderate agreement (kappa: 0.593, 95 % Confidence Interval: [0.58–0.61], *p*-value <0.001) and a significant correlation (Spearman: 0.772, 95 % Confidence Interval: [0.76–0.78], *p*-value <0.001).

**Table 2:** The risk for 10-years cardiac events assessed by FRS and ASCVDrs, N (%).

ASCVDrs	FRS			
	Total	Low risk	Intermediate risk	High risk
Low risk	5332 (59.4 %)	4833	499	0
Intermediate risk	1073 (11.9 %)	363	697	13
High risk	2576 (28.7 %)	322	860	1394
Total	8981 (100.0 %)	5518 (61.4 %)	2056 (22.9 %)	1407 (15.7 %)

ASCVDrs, Atherosclerotic Cardiovascular Disease Risk Score; FRS, Framingham Risk Score.

**Fig. 1:**
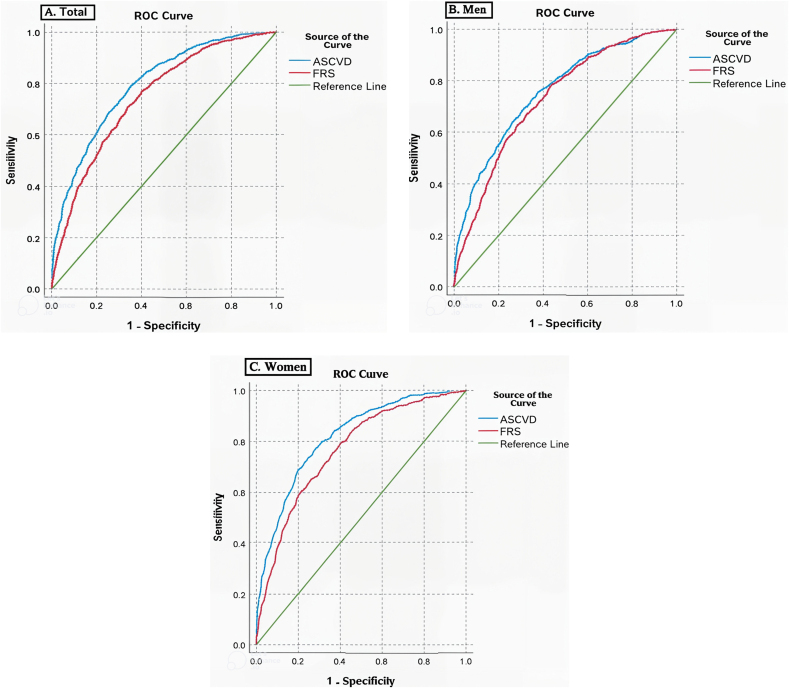
Performance of Framingham risk score and ASCVD risk score in total, men, women participants (from left to right).

Table 3 compares the performance of the Framingham Risk Score and ASCVDrs in the detection of high-risk individuals among men and women. The discrimination ability of both risk scores was acceptable and significantly higher in women (*p*-value <0.001). Compared to FRS, ASCVDrs had a significantly higher discrimination ability in both men and women. The ROC curves of ASCVDrs and FRS for total population, men, and women are depicted in Fig. 1. ASCVDrs had a higher sensitivity than FRS; however, FRS had a higher sensitivity in men. FRS had a higher specificity in comparison to ASCVDrs but lower specificity in men. ASCVDrs had higher PPV than FRS in both genders. Also, ASCVDrs had a higher NPV in comparison with FRS among women; whereas, it had a lower NPV among men. Optimal cut-off points are summarized in Table 3. Both risk prediction models had lower cut-off points in our population, and men had higher cut-off points.

**Table 3:** Comparing the efficiency of ASCVDrs and FRS in the detection of high-risk group among women and men.

Detection indices	ASCVDrs			FRS		
	Total	Men	Women	Total	Men	Women
AUC∗	0.794	0.758	0.817	0.746	0.730	0.764
Sensitivity	79.3 %	67.6 %	76.3 %	71.6 %	77.7 %	62.5 %
Specificity	64.5 %	70.3 %	72.8 %	65.1 %	56.7 %	82.5 %
PPV	36.3 %	43.9 %	33.6 %	34.4 %	38.1 %	27.3 %
NPV	92.4 %	86.4 %	94.4 %	90.0 %	88.2 %	93.4 %
Cut off	4.36 %	7.92 %	3.24 %	9.05 %	12.6 %	5.04 %

ASCVDrs, Atherosclerotic Cardiovascular Disease Risk Score; FRS, Framingham Risk Score; AUC, Area Under the Curve; NPV, Negative Predictive Value; PPV, Positive Predictive Value.

∗ *p*-value <0.001.

## 4 Discussion

The present study purposed to compare the discrimination ability of the ASCVDrs and FRS to find out which is the most appropriate for the Iranian population. Our findings revealed that the discriminative ability of both FRS and ASCVDrs was acceptable, but ASCVDrs significantly detected high-risk people better than FRS in both men and women. Both risk prediction models predict CVD events more accurately in women, which is in line with previous studies [11]. ASCVDrs also advantaged a higher sensitivity; however, it had 0.6 % lower specificity compared with FRS. Higher discrimination ability of ASCVDrs improves the performance in detecting high-risk patients, which leads to better prevention outcomes that, consequently, develop society's health and economy [21,22]. Furthermore, ASCVDrs classified more people as high-risk among our participants, which is agreed with previous studies [23,24]. Our data also suggest that FRS and ASCVDrs have a moderate agreement, higher than studies conducted in Nigeria [24] and Iran [18].

Additionally, our research has uncovered another intriguing result, which presented appropriate optimal cut-off points for each risk model. The specific cut-off point would engender the opportunity to predict the CVD risk more accurately and prevent over- or under-treatment [11]. Both optimal cut-off points were lower than the suggested ones by the prevailing guidelines [7,8]. Lower optimal cut-off points indicate that cardiac events could happen in people with fewer risk factors in comparison with other populations. Therefore, the Iranian population needs more precise and tight risk control. Additionally, lower cut-off points increase the number of individuals identified as high-risk, thereby expanding the pool of candidates eligible for lipid-lowering medication. Previous research has demonstrated that lower cut-off points may classify a greater percentage of individuals who experience cardiac events as high-risk [25]. Current evidence supports the idea that lower cut-off points for statin prescription might be cost-beneficial and decrease the risk of cardiac events [26,27].

Compatible with our findings, previous studies in Iran [18,28] showed that ASCVDrs made more people eligible for statin use in comparison with FRS. In 2017, a study of 3086 people in northern Iran showed that more participants were recommended to use statins with ASCVDrs compared to FRS (men: 58.2 % vs. 27.1 %; women: 39.7 % vs. 33.0 %) [28]. A recent study compared ASCVDrs and FRS among 289 obese Iranians (86.2 % women). ASCVDrs identified 14.9 % as high-risk, whereas FRS only identified 1 % as high-risk. This study also found a lower level of agreement between ASCVDrs and FRS in comparison to our study (Kappa: 0.236 vs. 0.593) [18]. Compared to our study, none of the aforementioned studies compared the discriminative ability, sensitivity, specificity, and optimal cut-off point.

ASCVDrs was developed based on the data from five cohorts with divergent races and ethnicities, including Framingham Original and Offspring Study cohorts [8,29], while FRS was developed only based on Framingham Original and Offspring Study cohorts [7]. Furthermore, race is one of the parameters of ASCVDrs calculation, whereas race is not involved in FRS calculation. All these differences make ASCVDrs a better risk prediction model than FRS; however, different studies showed controversial results in various populations [11]. Congruent with our findings, a study in a multiethnic population revealed that ASCVDrs was significantly a more accurate predictor of CVD than FRS (AUC [95 % CI] ASCVDrs: 0.737 [0.713, 0.762]; FRS: 0.717 [0.691, 0.743]) and replacing the ASCVDrs with FRS lead 17.1 % increase in statin prescription for CVD prevention [30]. Also, L. Pennells et al. analyzed the data driven from 22 countries consisting of 360,737 individuals and showed that ASCVDrs advantaged a greater discriminative performance than FRS [31]. Moreover, a prospective cohort study in Australia showed that ASCVDrs had a higher discrimination ability than FRS in both men and women [17]. In addition, a systematic review showed that ASCVDrs is superior to FRS in case of discrimination, and both have a higher discriminative ability in women; however, the results were heterogeneous because of diverse populations [11]. Contrary to our results, a prospective study in Malaysia among 12,573 participants indicated that FRS had a stronger discriminative performance than ASCVDrs in this Asian population (AUC [95 % CI] FRS: 0.750 [0.728, 0.772]; ASCVDrs: 0.546 [0.516, 0.576]) [32]. In a study on 1110 Indian patients on the day they experienced myocardial infarction, FRS and ASCVDrs labeled 51.9 and 28.3 % of them as high-risk [16].

Variations in the performance of conventional risk scores such as ASCVDrs and FRS across different populations are influenced by factors including levels of risk factors within the population, the relative risk of each risk factor, and the average population risk of CVD [33]. These differences can be attributed to genetic variations, environmental factors, and lifestyle. Lifestyle risk factors, such as inadequate physical activity and unhealthy dietary habits, not only impact established CVD risk factors but also contribute to oxidative stress, inflammation, and dysfunction of the endothelium [34]. Incorporating these lifestyle factors into risk assessment tools could enhance the accuracy of CVD risk prediction models [35,36]. Recent genome-wide association studies have identified genetic factors, specifically multiple single-nucleotide polymorphisms on the chromosome 9p21 region, that are associated with CVD risk [37]. Some experts have proposed using genetic risk scores to aid in the early identification of high-risk individuals. However, studies have demonstrated that the use of genetic risk scores only marginally improves the assessment of CVD risk compared to traditional risk scores [38,39]. This limited improvement is largely due to the fact that most genetic factors influence CVD risk by altering mechanisms related to blood pressure, glucose levels, and lipid regulation, which are already taken into account in conventional risk scores [38,39]. Additionally, challenges related to accessing genetic testing facilities and methodological complexities have restricted the widespread application of genetic factors in primary prevention strategies for CVD [38].

### 4.1 Strengths and limitations

Our study had some strengths. First, this study was the first study of its kind in the Iranian population that compared the ASVD and FRS using AUC and suggested new optimal cut-off points. Additionally, this study benefited a large sample size (nearly 9000 participants) from two reliable datasets. However, our study also had some limitations. First, the present study was cross-sectional, so we could not assess the actual 10-year CVD risk to compare with the predicted risk by risk scores. Also, we couldn't differentiate between intermediate and low-risk individuals due to the cross-sectional nature of our study. Therefore, further study is needed to compare these two CVD risk prediction models in a longitudinal study. Second, Iran is a multiethnic country; hence, our results might be controversial in some areas of Iran. So, future research is required to compare risk scores in different regions of Iran.

## 5 Conclusion

In conclusion, ASCVDrs is superior to FRS for the Iranian population in case of accurate discrimination of high-risk individuals. Also, the application of updated cut-off points strengthens this risk score. Since ASCVDrs makes more people eligible for treatment with antihypertensive drugs or statins, which decreases the CVD risk, it may increase over-medicalization. Further studies are recommended to evaluate the outcomes of utilizing ASCVDrs instead of FRS.

## Funding

This research did not receive any specific grant from funding agencies in the public, commercial, or not-for-profit sectors.

## CRediT authorship contribution statement

**Matin Sepehrinia:** Conceptualization, Methodology, Writing – original draft. **Hossein Pourmontaseri:** Conceptualization, Formal analysis, Methodology, Writing – original draft. **Mehrab Sayadi:** Formal analysis, Methodology. **Mohammad Mehdi Naghizadeh:** Formal analysis, Methodology, Writing – review & editing. **Reza Homayounfar:** Supervision, Writing – review & editing. **Mojtaba Farjam:** Data curation, Investigation, Resources. **Azizallah Dehghan:** Data curation, Investigation, Resources. **Abdulhakim Alkamel:** Conceptualization, Project administration, Supervision, Writing – review & editing.

## Declaration of competing interest

The authors declare no conflict of interest.
